# Alterations of the axon initial segment in multiple sclerosis grey matter

**DOI:** 10.1093/braincomms/fcac284

**Published:** 2022-11-04

**Authors:** Aysegul Dilsizoglu Senol, Giulia Pinto, Maxime Beau, Vincent Guillemot, Jeffrey L Dupree, Christine Stadelmann, Jonas Ranft, Catherine Lubetzki, Marc Davenne

**Affiliations:** Sorbonne University, Paris Brain Institute—ICM, Inserm, CNRS, Pitié-Salpêtrière Hospital, Paris, France; Sorbonne University, Paris Brain Institute—ICM, Inserm, CNRS, Pitié-Salpêtrière Hospital, Paris, France; Institut de Biologie de l’École Normale Supérieure (IBENS), École Normale Supérieure, CNRS, Inserm, PSL Research University, Paris, France; Sorbonne University, Paris Brain Institute—ICM, Inserm, CNRS, Pitié-Salpêtrière Hospital, Paris, France; Institut Pasteur, Université de Paris, Bioinformatics and Biostatistics Hub, Paris F-75015, France; Department of Anatomy and Neurobiology, Virginia Commonwealth University, Richmond, VA, USA; Hunter Holmes McGuire VA Medical Center, Richmond, VA, USA; Institute of Neuropathology, University Medical Center Göttingen, Göttingen 37075, Germany; Institut de Biologie de l’École Normale Supérieure (IBENS), École Normale Supérieure, CNRS, Inserm, PSL Research University, Paris, France; Sorbonne University, Paris Brain Institute—ICM, Inserm, CNRS, Pitié-Salpêtrière Hospital, Paris, France; Assistance Publique des Hôpitaux de Paris (APHP), Pitié-Salpêtrière Hospital, DMU Neurosciences, Paris, France; Sorbonne University, Paris Brain Institute—ICM, Inserm, CNRS, Pitié-Salpêtrière Hospital, Paris, France

**Keywords:** axon initial segment, multiple sclerosis, grey matter lesion, neocortex, cerebellum

## Abstract

Grey matter damage has been established as a key contributor to disability progression in multiple sclerosis. Aside from neuronal loss and axonal transections, which predominate in cortical demyelinated lesions, synaptic alterations have been detected in both demyelinated plaques and normal-appearing grey matter, resulting in functional neuronal damage. The axon initial segment is a key element of neuronal function, responsible for action potential initiation and maintenance of neuronal polarity. Despite several reports of profound axon initial segment alterations in different pathological models, among which experimental auto-immune encephalomyelitis, whether the axon initial segment is affected in multiple sclerosis is still unknown. Using immunohistochemistry, we analysed axon initial segments from control and multiple sclerosis tissue, focusing on layer 5/6 pyramidal neurons in the neocortex and Purkinje cells in the cerebellum and performed analysis on the parameters known to control neuronal excitability, i.e. axon initial segment length and position. We found that the axon initial segment length was increased only in pyramidal neurons of inactive demyelinated lesions, compared with normal appearing grey matter tissue. In contrast, in both cell types, the axon initial segment position was altered, with an increased soma-axon initial segment gap, in both active and inactive demyelinated lesions. In addition, using a computational model, we show that this increased gap between soma and axon initial segment might increase neuronal excitability. Taken together, these results show, for the first time, changes of axon initial segments in multiple sclerosis, in active as well as inactive grey matter lesions in both neocortex and cerebellum, which might alter neuronal function.

## Teaser

Dilsizoglu Senol et al. highlight the importance of the axon initial segment in multiple sclerosis by demonstrating its shift away from the soma in both the neocortex and the cerebellum of multiple sclerosis active and inactive lesions and by showing evidence that this change could alter neuronal function.

## Graphical Abstract

**Graphical Abstract:**
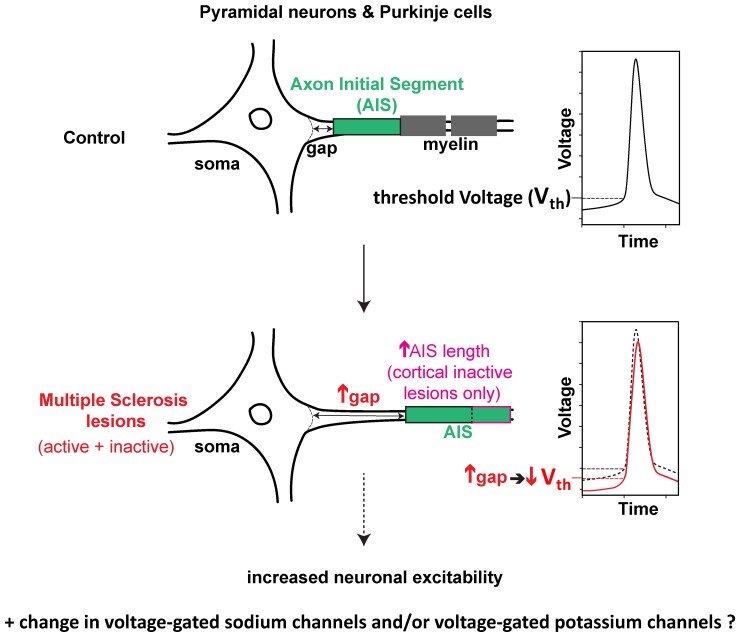

## Introduction

It is now well established that in multiple sclerosis (MS), central nervous system pathology exceeds white matter, and that grey matter damage occurs early in disease evolution, correlating with clinical disability and cognitive dysfunction.^1–4^ Associated with an inflammatory component consisting of both innate and adaptive immunity, grey matter damage combines structural and functional changes such as demyelination, neuritic transections and neuronal loss^5,6^ as well as synaptic pathology.^7,8^ Whether the axon initial segment (AIS), a key player in neuronal function, is altered in MS grey matter is unknown.

Located next to the soma and immediately followed by the myelin sheath in most neurons, the AIS is the site of action potential (AP) initiation. This is due to the dense aggregation of voltage-gated sodium (Nav) channels by the cytoskeleton-linked anchoring protein, AnkyrinG (AnkG), which also clusters particular voltage-gated potassium (Kv) channels.^9^ This molecular architecture is very similar to that of nodes of Ranvier, which have been shown to be altered in MS white matter plaques.^10,11^ The AIS also acts as a barrier between the somatodendritic and axonal compartments due to an AIS-specific AnkG-organized cytoskeleton and plasma membrane composition, and as such maintains axonal integrity and neuronal polarity.^12,13^ AnkG is considered as the AIS master organizer protein and its expression along the AIS is thus critical for allowing the AIS to play its two key roles, spike initiation and maintenance of axonal identity. Indeed, AnkG loss leads to a total dismantling of AIS-constituent proteins, including Nav channels, failure of spike initiation and loss of axonal identity.^14–17^

The AIS is a plastic domain: its length, its position relative to the soma and/or its ion channel composition can vary, depending for instance on the level of input activity received by the neuron.^18^ These AIS changes allow the neuron’s excitability properties to be modulated and in some cases, homeostatically fine-tuned to the neuron’s environment.^18^ The AIS is also a vulnerable domain whose properties have been shown to be altered in many pathological conditions, as in Alzheimer’s disease models^19–21^ in epileptic syndromes,^22,23^ in an Angelman syndrome mouse model,^24^ or upon ischemia^25^ or traumatic brain injury^26^ in mice. To our knowledge, AISs have not been analysed in MS tissue, although AIS alterations have been reported in experimental models of MS. In the inflammatory myelin oligodendrocyte glycoprotein-induced experimental allergic encephalomyelitis (EAE) mouse model, major AIS changes such as reduced length and AIS loss were found.^27^ In contrast, such changes were not detected in the (non-inflammatory) cuprizone-induced demyelination mouse model^27,28^ where a change in AIS position (AIS onset closer to the soma) impacting excitability properties was reported.^28^ These experimental results paved the way for analysing AISs in MS tissue. Here, we show that AISs start further away from the soma in both cortical pyramidal neurons and cerebellar Purkinje cells in demyelinated grey matter lesions, whereas AIS length is changed only in pyramidal neurons of inactive lesions. Furthermore, using a computational model, we provide evidence that this enlarged soma-AIS gap could potentially increase neuronal excitability.

## Materials and methods

### Human post-mortem samples

Tissue samples and associated clinical and neuropathological data (Table 1) were supplied by the Multiple Sclerosis Society Tissue Bank funded by the Multiple Sclerosis Society of Great Britain and Northern Ireland, registered charity 207495. The MS and Parkinson’s Tissue Bank at Imperial College London has been approved as a Research Tissue Bank by the Wales Research Ethics Committee (Ref. No. 18/WA/0238). As a part of this ethical approval, the Tissue Bank sought generic ethical approval on behalf of researchers using tissue or data supplied by the bank.^29^

**Table 1:** Patients characteristics

Case	Age	Sex	CNS pathology	MS duration (years)	MS phenotype	Post-mortem delay (hours)
CO1	35	M	None	–	–	22
CO2	78	F	mild aging-related changes	–	–	33
CO3	60	F	diffuse hypoxic changes	–	–	13
CO4	88	M	aging-related changes	–	–	22
CO5	68	M	aging-related changes	–	–	30
CO6	84	M	aging-related changes	–	–	5
CO7	68	M	micro vascular pathology	–	–	10
MS1	49	F	MS	14	PPMS	24
MS2	44	M	MS	unknown	SPMS	16
MS3	44	F	MS	16	SPMS	18
MS4^a^	45	F	MS	6	RRMS	28
MS5	52	F	MS	20	SPMS	7
MS6	88	F	MS	30	PPMS	22
MS7	44	F	MS	unknown	SPMS	9
MS8	57	F	MS	unknown	SPMS	13
MS9^a^	51	F	MS	unknown	SPMS	10
MS10	35	F	MS	5	SPMS	9
MS11	43	M	MS	18	SPMS	26
MS12	42	M	MS	27	PPMS	20

CO = control case; MS = Multiple sclerosis case; RRMS = relapsing-remitting MS; SPMS = secondary progressive MS; PPMS = primary progressive MS. ^a^Normal appearing grey matter (NAGM), without cortical plaque (cerebellar sample only).

Despite many attempts, we were unable to visualize AISs (with antibodies directed against AIS proteins, such as AnkG or Nav) on paraffin-embedded sections. We therefore undertook our study on snap-frozen tissue. After post-mortem delays ranging from 5 to 24 h, brains had been dissected out, immediately thereafter cut in 1 cm-thick coronal slices then into 2 × 2 × 1 cm^3^ blocks, before being frozen by immersion into −50°C isopentane and stored at −85°C.

Table 1 shows the characteristics (age, sex, disease phenotype and disease duration) of the 12 MS cases and seven control cases used in this study. As shown in Table 2, one to seven tissue blocks were available for each MS case. They consist of both neocortical and/or cerebellar cortical samples, characterized as normal-appearing grey matter (NAGM), active and inactive lesions (see Supplementary Figs. 1 and 2).

**Table 2:** List of tissue samples (marked by *) used in this study, showing their control or MS case of origin and the tissue category they contained (CO = control tissue/case; NAGM = normal appearing grey matter)

	Neocortex				Cerebellar cortex			
Tissue category	CO	NAGM	Active lesion	Inactive lesion	CO	NAGM	Active lesion	Inactive lesion
CO1	*				*			
CO2	*							
CO3	*							
	*							
CO4	*				*			
CO5	*							
	*							
CO6					*			
CO7					*			
MS1		*		*				*
MS2		*		*		*	*	
MS3		*	*	*			*	*
		*						*
MS4						*		
MS5		*	*	*		*	*	
MS6		*		*				*
MS7		*		*				*
MS8		*		*		*		
		*						
MS9						*		
						*		
MS10		*	*					
		*	*					
				*				
MS11		*		*				
		*		*				
MS12		*		*				

Each line corresponds to a different neocortex or cerebellar cortex tissue block.

### Immunolabelling

***(i) For lesion characterization***

Tissue blocks were cut into 20 μm thick cryostat sections. Once dried, sections were washed (in phosphate buffer saline, PBS) and fixed in 4% paraformaldehyde (PFA) for 40 min. After washing, sections were first incubated for 30 min in pure methanol and 2% H_2_O_2_(for MHC II immunolabelling), or (after immersion for 20 min at −20°C in pure ethanol and washes) for 5 min in 40% MeOH and 1% H_2_O_2_ (for proteolipid protein, PLP, immunolabelling). A blocking step was then performed: 1 h with 4% BSA and 0,1% Triton X-100 (for MHC II), or 1 h with 10% normal goat serum (Gibco BRL) and 0,4% Triton X-100 (for PLP). Slides were then incubated overnight at 4°C with primary antibodies diluted in the respective blocking solution (for PLP, 0,4% Triton was replaced by 0,2% Triton). After washing, slices were incubated for 45 min with biotinylated secondary antibodies (Vectastain kit) diluted in PBS, washed, treated for 1 h with the Biotin-Avidin solution (Vectastain kit) and revealed with the DAB chromogen (25 mg/50 ml), mixed with 25% Tris-base Buffer 1M and H_2_O_2_ (4.0E-3% for MHC II or 8.0E-3% for PLP) and nickel ammonium sulphate at 100 mg/50 ml (for MHC II only). Slides were finally dehydrated into increasing ethanol concentrations (30 to 100%) washed in Xylene and mounted with Eukit. After MHC II immunolabelling, Luxol fast blue (LFB) staining was performed before dehydration: sections were immersed in 70% ethanol, incubated overnight with LFB at 60°C, rinsed in 95% ethanol followed by water, and a good contrast was achieved by repeated cycles of incubation in lithium carbonate (0,5 g/l), followed by rinses in 70% ethanol and water. Bright field (BF) mosaic images of tissue sections labelled for PLP and MHC Class II and LFB were taken with a 5 × objective in order to visualize the whole tissue section and used as references to decide where to focus for AIS analysis acquisitions.

MHC II density quantification:

From these BF mosaic images of LFB and MHC II-labelled sections described above, the density of MHC II-positive cells was quantified. A rectangular region of interest (ROI) was selected (corresponding to the purple panels in Supplementary Figs. 1 and 2, respectively) and its area measured with Image J (NIH ImageJ software; Bethesda, MD, USA); the ROI size was kept constant across all four tissue categories. With ImageJ’s colour threshold tool, the total area occupied by all MHC II-positive cells within the ROI was measured for each tissue category and reported as a percentage of the total ROI area.

***(ii) For AIS analysis***

AIS analysis was performed on sections adjacent to those used for tissue characterization. Dried sections were washed in PBS and fixed for 5 min in 2% PFA (in PBS, as for the following incubations). After washing followed by a blocking step in 10% NGS and 0.4% Triton, sections were incubated overnight at 4°C with primary antibodies in 10% NGS and 0.2% Triton. Slides were then washed and incubated for 2 h with secondary antibodies diluted in the same solution as for primary antibodies. Finally, slides were washed and mounted with Dapi-Fluoromount-G (SouthernBiotech). When PLP immunolabelling was added, prior to mounting, a second fixation step (5 min in 4% PFA, followed by washing and incubation for 15 min at −20°C in pure ethanol) was performed before incubation with the primary and secondary antibodies as before.

### Antibodies

The primary antibodies used were the following: anti-AnkG (mouse IgG2a, Neuromab, clone N106/36, 1:200), anti-PLP (hybridoma of polyclonal rat IgGs, clone AA3, gift from K. Ikenaka, University of Okazaki, Japan, 1:10), anti-Human HLA-DP, DQ, DR (‘anti-MHC II’, mouse IgG1, Dako clone CR3/43, 1:150), anti-Calbindin (mouse IgG1, Sigma-Aldrich, CB-955, 1:500), anti- non-phosphorylated neurofilament H (‘anti-SMI-32’, mouse IgG1, BioLegend clone SMI32, 1:1000). The secondary antibodies used were the following: Alexa 488/Alexa 594 (both 1:1000)/Alexa 647 (1:500)-conjugated secondary antibodies (Invitrogen; Villebon sur Yvette, France) directed against rabbit/rat polyclonal or mouse monoclonal primary antibodies.

### AIS length and soma-AIS gap measurements

AIS analysis was performed by immunolabelling with AnkG and SMI32 (pyramidal neuron marker) in the neocortex or with AnkG and Calbindin (Purkinje cell marker) in the cerebellum. PLP immunolabelling was also performed to further confirm the demyelination status of lesions analysed. Neocortical SMI32-positive pyramidal neurons belonging to layer 5/6 were identified thanks to their typical large pyramidal-type soma, their thick apical dendrite and their position next to the white matter—grey matter border. Cerebellar Purkinje cells were easily identified with Calbindin immunolabelling. AnkG-immunolabeled AISs from these two neuronal populations were analysed with an Axiovert 200M (Carl Zeiss) fluorescence microscope, equipped with an Apotome module. Images were acquired with the Axiocam MRm camera (Carl Zeiss) using the Axiovision (Carl Zeiss) image analysis software, using a 20 × (0.8 NA) and a 40 × oil (0.75 NA) objective for cerebellum and cortex sections, respectively.

AIS length analysis was performed by tracing the AIS in three dimensions with the Simple Neurite Tracer plugin of Image J, whereas soma-AIS gap analysis was performed by tracing the soma-AIS gap in two dimensions with the Image J line tracing tool from the axon start position along the soma contour to the AIS start position. It turned out that analysed AISs were quite parallel to image planes, such that in three dimensions, the axon start site was never more than two image planes apart from the AIS start position. With such a small inclination angle relative to image planes (the step size between image plane was 0.5 µm for the cortex and 0.55 µm for the cerebellum) and the fact that soma-AIS gap traces were always found to be linear, the bias introduced by measuring soma-AIS gaps in two dimensions rather than in three dimensions could be considered as negligeable.

For both, Z-stack images were zoomed-in and carefully traced from or to the beginning of the AIS. The AIS start position was defined with the help of the AnkG immunolabelling, which draws a cone shape at the beginning of the AIS, following the axon hillock. Throughout our analyses, the AIS start position was defined as being the bottom of this cone or, when the cone shape was not obvious, the first AnkG+ pixel at the beginning of the AIS in the middle of the axon.

AIS length measurements in three dimensions were restricted to fully intact AISs having clear SMI32 or Calbindin labelling right before and after AnkG labelling or AISs where the typical axon hillock could be clearly observed. The integrity of AISs was further verified in three dimensions by checking and comparing both channels (AnkG and either SMI32 or Calbindin) in each image plane throughout the volume of the analysed image and incomplete AISs were not analysed. As for the soma-AIS gap analysis, we analysed AISs either affixed to a pyramidal neuron or a Purkinje cell soma or AISs present on a SMI32+ or Calb+ axon connected to its respective soma. Thus, AISs cut distally were also considered. Length and soma-AIS gap measurements were performed blinded with respect to the tissue category of origin. We have analysed on average two sections per tissue block for both AIS length and soma-AIS gap measurements in cortex and cerebellum (except for cortical active lesions, where we analysed four and three sections for length and gap, respectively, in order to increase the number of analysed AISs).

### Statistical analysis

For each AIS, length and soma-AIS gap measures were plotted as individual dots, where each colour corresponds to a different tissue block. Both mean and standard error of the mean for each tissue category were calculated. For all these measures (AIS length, soma-AIS gap), to take the intra-individual variability into account, linear mixed-effect models were used, in which the individual factor was considered a random effect. After building the model, a *post hoc* procedure was applied to assess the difference between each pair of tissue categories, a *P*-value was therefore computed for each of these pairs and corrected with a Tukey procedure (and reported as such in the results section).

Note that given the atypical distribution of soma-AIS gap values, *P*-values were computed through permutations (10 000 permutations), followed by a Bonferroni correction for multiple comparisons.

### Model description and simulations protocols

To simulate the neocortical pyramidal cell activity, we used a previously published model available on modelDB (http://modeldb.yale.edu/114394), implemented for the NEURON simulation environment. To simulate the Purkinje cell activity, we used a multi-compartmental model previously developed^30^ also available on modelDB (http://modeldb.yale.edu/229585) and implemented for the NEURON simulator’s Python interface. Both models are described in Supplementary material.

### Data availability

The authors confirm that the data supporting the findings of this study are available within the article, its Supplementary material and/or from the corresponding author.

## Results

### Characterization of MS grey matter lesions

To study AISs in MS tissue, we first characterized grey matter lesions found in cortical and cerebellar tissue blocks from MS cases’ brains. Sections were processed with: (i) LFB staining (histological myelin stain), to ensure the discrimination between white and grey matter, combined on the same tissue section with MHC II immunolabelling, to detect inflammatory cells; and (ii) PLP immunolabelling (myelin marker) on an adjacent section, to allow, better than LFB staining, the discrimination within the grey matter between demyelinated lesions and normally myelinated areas (NAGM and control tissue) (Supplementary Figs. 1 and 2). Tissues were then categorized as either: (a) ‘control’ tissue (from non-MS cases), where myelin could be detected both with LFB staining and PLP labelling and no or only a few MHC+ cells could be found (Supplementary Figs. 1A & E and 2A & E), or (from MS cases) (b) ‘NAGM’, with similar myelinated aspect as in control tissue (Supplementary Figs. 1B and 2B), (c) ‘active lesion’, where a clear demyelination and a high density of MHC+ cells were detected (Supplementary Figs. 1C, E and 2C, E, lesion borders are indicated by dashed lines), (d) ‘inactive lesion’, where clear demyelination was detected, with the absence or a low density of inflammatory MHC+ cells (Supplementary Figs. 1D & E and 2D & E, lesion border is indicated with a dashed line for the cortical inactive lesion but not for the cerebellar inactive lesion, as the adjacent subregion is devoid of myelin).

### Neocortical layer 5/6 pyramidal neurons have an altered AIS position in both active and inactive MS lesions

We first analysed the structural properties of AISs known to control the neuron’s spiking properties, namely the length and position of AISs.^31–33^ Given the variability of AIS length between different cortical neuronal subtypes, we focused our analysis on pyramidal neurons (labelled with SMI-32 antibody) belonging to cortical layers 5 and 6. Furthermore, for statistical analyses, linear mixed-effect models in which the individual factor was considered a random effect were used, to further eliminate a potential bias due to intra-individual variability (see Materials and Methods).

To ensure the accuracy of (AnkG-labelled) AIS length and position (soma-AIS gap) measurements, the analysis was restricted to fully intact AISs (with SMI-32-positive axon hillock and axonal segments encompassing AnkG labelling, see Materials and Methods for details) but also to AISs connected to their SMI-32 or Calbindin positive cell body. The number of such AISs that could be analysed was further reduced due to the use of snap-frozen tissue, which not only limited the thickness of sections but also often compromised the integrity of the tissue (hence of AISs).

**Figure 1:**
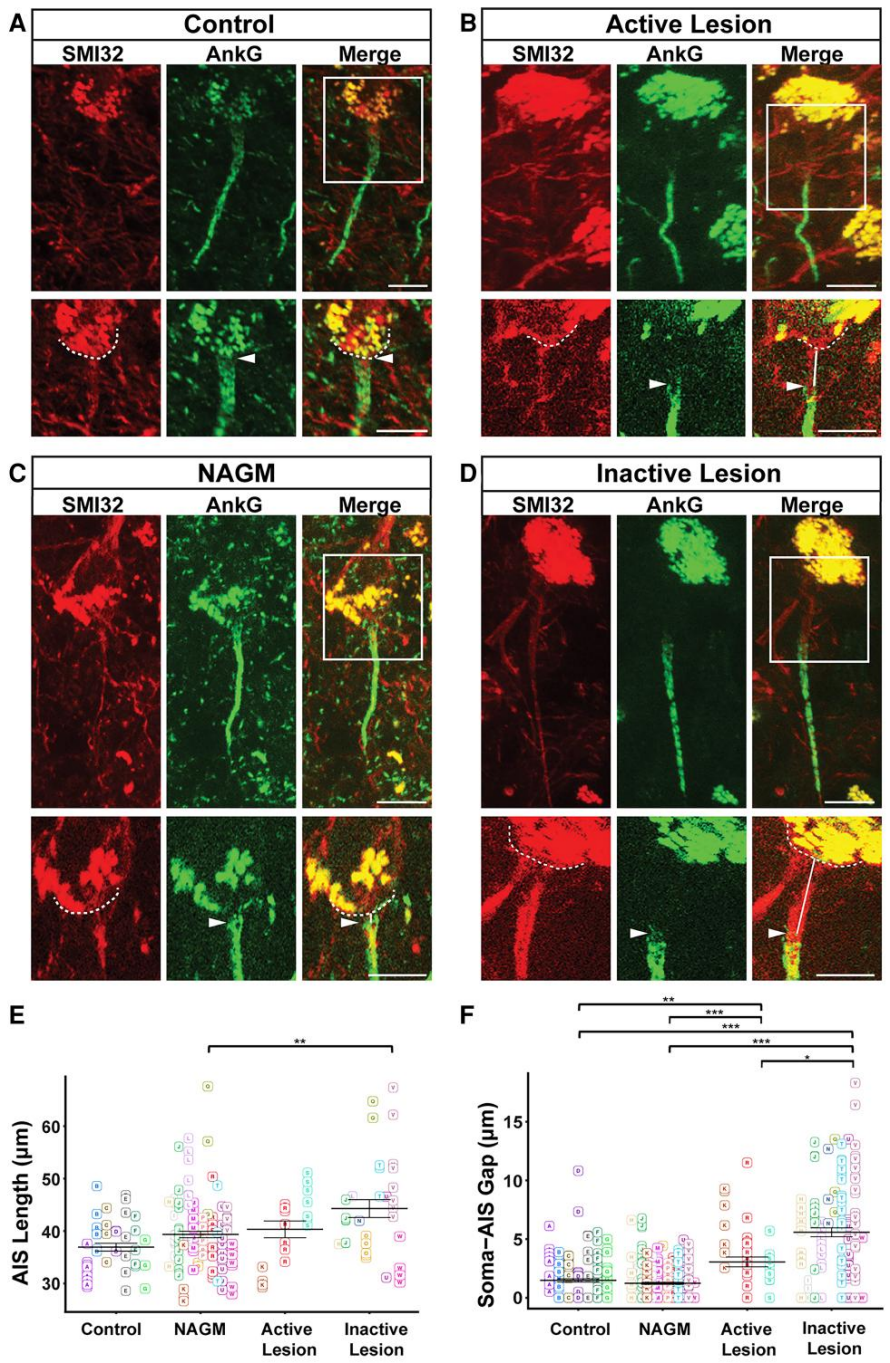
**AIS length and soma-AIS gap analysis in neocortical layer 5/6 pyramidal neurons.** (**A**) Representative cortical layer 5/6 pyramidal neurons from control, (**B**) active MS lesion, (**C**) NAGM, (**D**) inactive MS lesion tissues immunolabelled with anti-SMI32, and their AIS immunolabelled with anti-AnkG. A higher magnification of the frames indicated with the white rectangles in the merged channel panels are presented in the lower panels for each tissue category. Dashed lines indicate the contour of the soma, arrowheads indicate the start of the AIS, and white lines represent soma-AIS gaps. (**E**) AIS length and (**F**) soma-AIS gap measures were plotted in the presented graphs, where each AIS is identified by a square and each tissue block from which this AIS is coming from is identified by capital letter associated with a colour. For each tissue category, the mean AIS length ± SEM and the mean soma-AIS gap ± SEM is represented. A pairwise *t*-test with a Bonferroni correction for multiple testing was used together with linear mixed-effect models to take the intra-individual variability into account. *P*-values computed for each pair of tissue categories were corrected with a Tukey procedure. Brackets highlight statistically significant differences with * for 0.01 < *P* < 0.05; ** for 0.001 < *P* < 0.01; *** for *P* < 0.001. Scale bar: 10 μm. Image stacks were acquired with a 40 × objective; maximum intensity projection images of various sub-stack sizes (for upper and lower images of each panel) were used in order to best illustrate either the entire AIS with its soma (large sub-stacks) or just the beginning of the AIS and the soma contour (small sub-stacks), respectively.

AIS length and soma-AIS gap were compared between control tissue, MS NAGM, active MS lesions and inactive MS lesions (Fig. 1A–D). As shown on Fig. 1E, the mean length of AISs was: 36.92 ± 0.74 μm for control tissue (mean ± standard error of the mean (SEM), *n* = 45 AISs from 5 cases); 39.33 ± 0.62 μm for NAGM (*n* = 114 AISs from 10 cases); 40.31 ± 1.60 μm for active lesions (*n* = 18 from 2 cases) and 44.27 μm ± 1.69 μm for inactive lesion (*n* = 33 from 9 cases). No statistically significant difference between any pair of tissue categories was found, except for inactive lesions, whose AISs were found to be significantly longer than in NAGM (Fig. 1E). Note that active lesions suffer from a rather low number of AISs that could be analysed, in addition with disparate length measures, which make any conclusion about these neurons’ AIS length difficult to draw.

To analyse whether AIS position was affected in MS tissue, we used the same image acquisitions of AnkG+ AISs from SMI-32+ layer 5/6 pyramidal neurons. SMI-32 labelling allowed to precisely determine the outline of pyramidal neurons somata (dashed lines in Fig. 1A–D; note the strong superimposing autofluorescence of somatic lipofuscin accumulation), therefore, to measure the distance between the soma and the beginning of the AIS (pointed by the arrowhead in Fig. 1A–D), the soma-AIS gap. AISs were either directly apposed or very close to the soma in both control tissue and NAGM (mean soma-AIS gap, respectively: 1.48 ± 0.37 μm; *n* = 167 AISs from 5 cases; and 1.24 ± 0.09 μm; *n* = 261 AISs from 10 cases; Fig. 1F). In contrast, the mean soma-AIS gap was significantly increased in both active lesions (3.05 ± 0.42 μm; *n* = 44 AISs from 2 cases) and inactive lesions (5.58 ± 0.37 μm; *n* = 122 AISs from 9 cases), compared with control tissue or NAGM (Fig. 1F). The mean soma-AIS gap in inactive lesions was also significantly increased compared with that in active lesions.

We also plotted AIS length and soma-AIS gap measures from pyramidal neurons as a function of the patient of origin (Supplementary Fig. 3A and B). This plot further shows that because of a too small number of patients from which active lesions could be analysed (in addition with disparate AIS length measures), the AIS length analysis in active lesions could not be conclusive. However, the significant length change observed in inactive lesions compared with NAGM (Fig. 1E) was supported by the consistency of this result obtained from a relevant number of patients.

Altogether these results demonstrate that in neocortical pyramidal neurons soma-AIS gap was increased in active and inactive demyelinated lesions, whereas AIS length was increased only in inactive lesions.

### Cerebellar Purkinje cells have an altered AIS position in both active and inactive MS lesions

**Figure 2:**
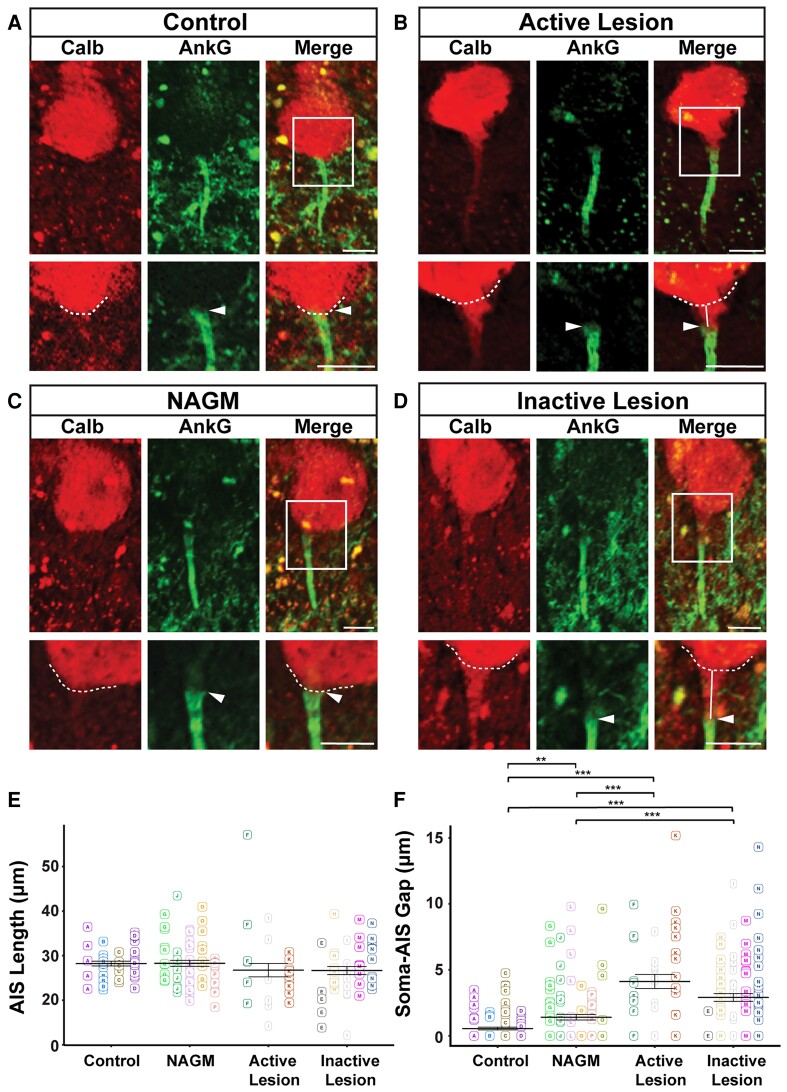
**AIS length and soma-AIS gap analysis in cerebellar Purkinje cells.** (**A**) Representative Purkinje cells from control, (**B**) active MS lesion (**C**) NAGM (**D**) inactive MS lesion tissues immunolabelled with Calb and AnkG were presented. A higher magnification of the frames indicated with the white rectangles in the merged channel panels are presented in the lower panels for each tissue category. The dashed line indicates the contour of the cell body, arrowheads indicate the start of the AIS, and white lines represent the soma-AIS gaps. (**E**) AIS length and (**F**) soma-AIS gap measures were plotted in the presented graphs, where each AIS is identified by a square and each tissue block from which this AIS is coming from is identified by capital letter associated with a colour. For each tissue category, the mean AIS length ± SEM and the mean soma-AIS gap ± SEM is represented. A pairwise *t*-test with a Bonferroni correction for multiple testing was used together with linear mixed-effect models to take the intra-individual variability into account. *P*-values computed for each pair of tissue categories were corrected with a Tukey procedure. Brackets highlight statistically significant differences with * for 0.01 < *P* < 0.05; ** for 0.001 < *P* < 0.01; *** for *P* < 0.001. Scale bar: 10 μm. Image stacks were acquired with a 20 × objective and maximum intensity projection images of various sub-stack sizes (for upper and lower images of each panel) were used in order to best illustrate either the entire AIS with its soma (large sub-stacks) or just the beginning of the AIS and the soma contour (small sub-stacks), respectively.

To investigate whether other neuronal populations display similar types of changes in terms of AIS structural characteristics, we analysed cerebellar Purkinje cells, given their important role in motor coordination and the frequent occurrence of cerebellar grey matter lesions in MS.^3^ Cerebellar control tissue, NAGM, active and inactive lesions were characterized as previously described. Purkinje cells were labelled with an anti-Calbindin antibody and AISs were labelled as described above, allowing Purkinje cell AIS lengths and soma-AIS gaps to be compared between control tissue, MS NAGM, active MS lesions and inactive MS lesions (Fig. 2A–D), using the same approach as the one used for pyramidal neurons.

As shown on Fig. 2E, the mean length of AISs was: 28.23 ± 0.54 μm for control tissue (*n* = 43 AISs from four cases); 28.28 ± 0.67 μm for NAGM (*n* = 62 AISs from five cases); 26.74 ± 1.50 μm for active lesions (*n* = 28 AISs from three cases); and 26.66 ± 0.90 μm for inactive lesion (*n* = 43 AISs from four cases). No statistically significant difference between any pair of tissue categories was found (Fig. 2E).

To analyse whether AIS position was affected in MS tissue, the same image acquisitions of AnkG+ AISs from Calb+ Purkinje cells were used, and soma-AIS gap was measured. Calbindin labelling allowed to precisely determine the outline of Purkinje cells somata (dashed lines on Fig. 2A–D), therefore, to measure the distance between the soma and the beginning of the AIS (pointed by the arrowhead in Fig. 2A–D), the soma-AIS gap. Whereas AISs were almost affixed to the soma in control tissue (mean soma-AIS gap: 0.55 ± 0.10 μm, *n* = 100 AISs from four cases), a short gap was observed in NAGM (mean soma-AIS gap: 1.41 ± 0.22 μm, *n* = 110 AISs from five cases; Fig. 2F). In contrast, a significant increase of soma-AIS gap was evidenced in both active lesions (4.12 ± 0.54 μm, *n* = 41 AISs from three cases) and inactive lesions (2.91 ± 0.29 μm; *n* = 105 AISs from four cases) compared with control tissue or NAGM (Fig. 2F).

Altogether these results show that AIS position but not length was altered in both active and inactive cerebellar demyelinated lesions.

As for cortical lesions, we also plotted AIS length and soma-AIS gap measures from Purkinje cells as a function of the patient of origin (Supplementary Fig. 3C and D). This plot shows that a good enough number of patients show consistent results, which support the former conclusions drawn about both AIS length and soma-AIS gap in Purkinje cells.

Taken together, this morphological analysis of AISs from neocortical layer 5/6 pyramidal neurons and from cerebellar Purkinje cells shows that AIS length is changed (increased) only in pyramidal neurons of inactive demyelinated MS lesions compared with NAGM. In contrast, soma-AIS gap is increased in both active and inactive lesions, compared with both control tissue and NAGM, changes that may lead to altered excitability properties.

In order to investigate whether there was any correlation between the distribution or the significant changes between tissue categories of AIS length or soma-AIS gap measures in pyramidal neurons or Purkinje cells and the disease duration, the age of the patient or the post-mortem delay before the brain sample was collected and frozen (as reported in Table 1), these measures were plotted as a function of each one of these variables (Supplementary Fig. 4). This representation did not reveal any such correlation.

### Functional consequences of AIS changes occurring in active demyelinated MS lesions, assessed by computational modelling

**Figure 3:**
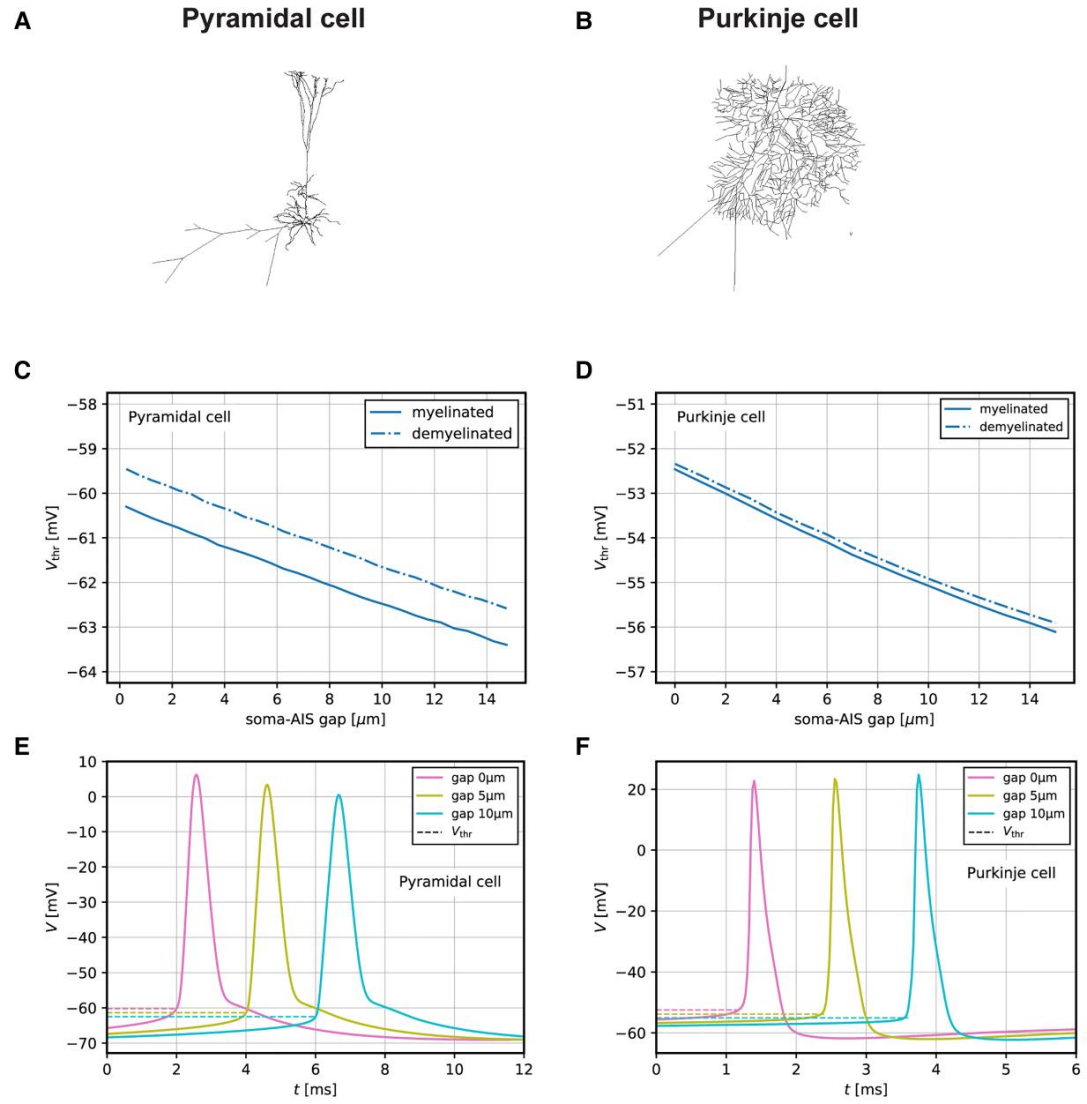
**Modelling of the functional consequences of soma-AIS gap changes observed in active demyelinated MS lesions in pyramidal and Purkinje cells. (**A**) Morphology sketch of pyramidal model cell.** (**B**) Morphology sketch of Purkinje model cell. (**C**) Variation of the AP voltage threshold with the length of the soma-AIS gap in pyramidal model cell. (**D**) Variation of the AP voltage threshold with the length of the soma-AIS gap in Purkinje model cell. (**E**) Example spiking traces of pyramidal model cell for different values of the soma-AIS gap. (**F**) AP traces of Purkinje model cell for different values of the soma-AIS gap.

Biophysical analyses of spike initiation in neurons highlighted the role of AIS geometry on neuronal excitability.^31,34^ To assess the potential effect of the increased soma-AIS gap observed in active and inactive demyelinated MS lesions, we simulated and analysed the excitability properties of both a model neocortical pyramidal cell (Fig. 3A) and a model Purkinje cell (Fig. 3B), based on detailed morphological reconstructions.^30,35^

For the pyramidal cell, we found that the AP voltage threshold decreases with increasing soma-AIS gap (−0.28 mV/µm, Fig. 3C and E, solid line). This effect persists and is comparable when the demyelinated status of the axon is taken into account (Fig. 3C, dashed line), which depolarizes the threshold by about 0.8 mV.

For the Purkinje cell, we similarly found that the AP voltage threshold decreases with increasing soma-AIS gap (−0.25 mV/µm, Fig. 3D and F, solid line), while demyelination alone leads to a very minor (∼0.1 mV) depolarization of the threshold (Fig. 3D, dashed line).

Overall, these results show that the increased soma-AIS gap can cause AP voltage threshold decrease in both neocortical pyramidal neurons and Purkinje cells, consistent with theoretical predictions for neurons in the ‘resistive coupling regime’.^31,34^ Under the assumption that other parameters that might affect spike threshold do not change, the observed changes in the soma-AIS gap may thus cause both of these neurons to be more excitable, i.e. more prone to spike, than in control tissue.

## Discussion

Grey matter damage is now established as an important contributor to long-term disability in MS. Grey matter atrophy results from a combination of demyelination, neurite transection and loss, as well as neuronal loss, partially related to inflammation.^36^ In addition, other alterations at the synaptic level have been described, such as reduction in synaptic density^7,37^ or dendritic spine loss.^8^ Although reduced synaptic density was mainly reported within cortical demyelinated lesions and the role of both demyelination and inflammation has been suggested, dendritic spine loss has been found both in lesions and NAGM, favouring the idea of a diffuse alteration partially independent of demyelination.

**Figure 4:**
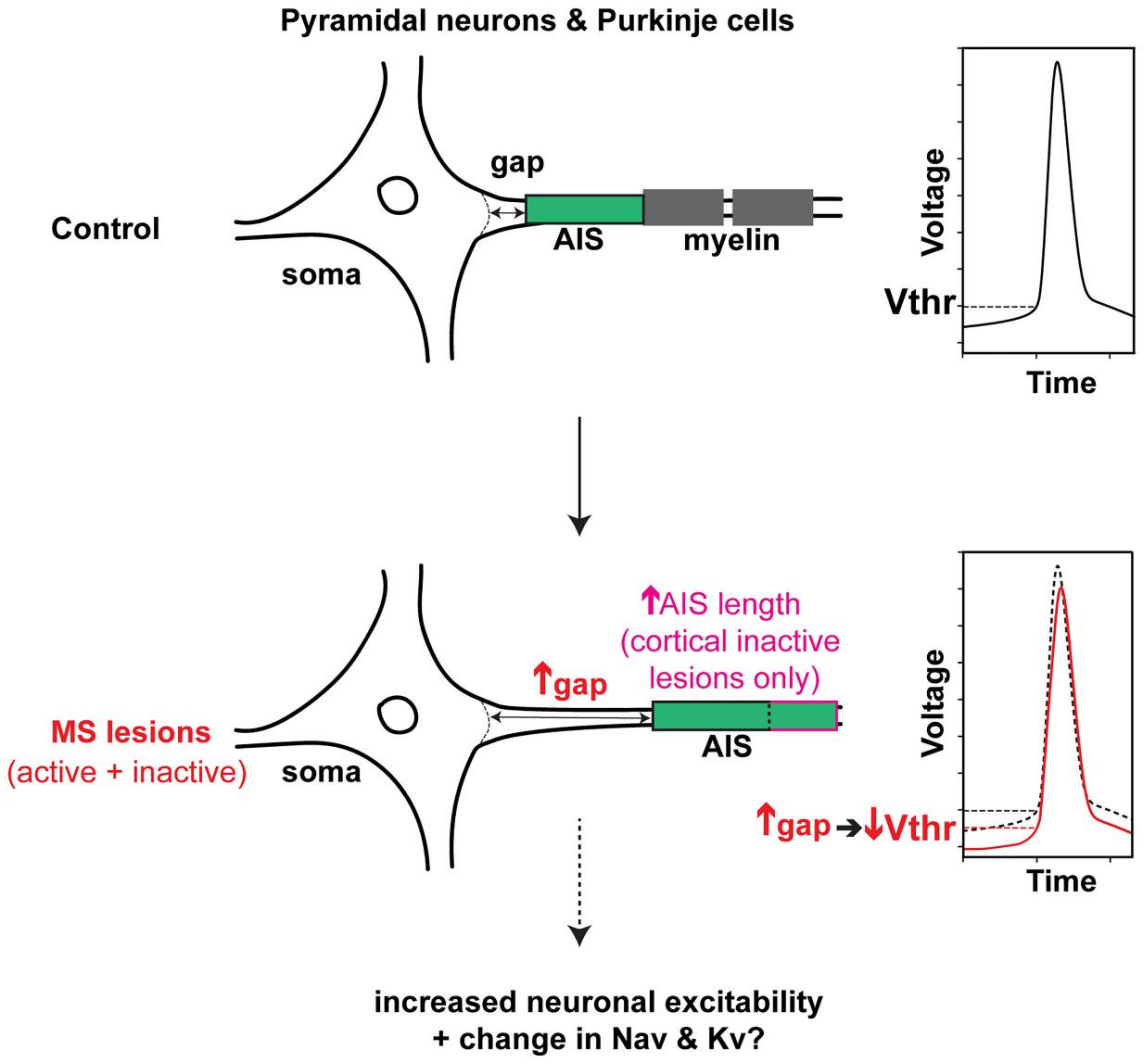
**Schematic drawing summarizing the main AIS changes observed in MS tissue.** Soma-AIS gap was found to be increased in demyelinated active and inactive MS lesions compared with control tissues for both pyramidal neurons and Purkinje cells. AIS length was also found to be increased, yet only in inactive lesions and for pyramidal neurons (were the sample size larger, such a change taking place also in active lesions should not be rule out). The increased soma-AIS gap was shown by modelling analyses to cause a decrease in the AP voltage threshold (Vthr), thus an increase in neuronal excitability. Together with potential accompanying changes in AIS Navs and Kvs, these AIS structural changes may play an important role in neuronal function alterations in MS.

Despite its major role in neuronal function, whether the AIS is affected in MS had not been addressed. In this context, AIS alterations have been reported in both cuprizone^27,28^ and EAE mouse models of MS.^27^ Here, we demonstrate for the first time that in MS the distance between soma and AIS is increased, for both neocortical pyramidal neurons and cerebellar Purkinje cells, in active as well as inactive demyelinated lesions, while the AIS length is changed (increased) only for pyramidal neurons of inactive lesions compared with NAGM. In addition, we provide computational evidence that this increased soma-AIS gap could lead to a decreased AP voltage threshold, which would make these neurons more prone to spike (Fig. 4).

This study of AISs in MS post-mortem tissue was challenging as several factors reduced the population size of AISs analysed. We overcame the difficulty of immunolabelling AISs on paraffin-embedded sections by working on snap-frozen samples, where immunolabelling was reliable and reproducible. However, tissue quality and limitation of section thickness (the best results were obtained with 20 µm) reduced the number of fully intact AISs particularly for the three-dimensional length analysis. In addition, obtaining cortical samples containing large enough active MS lesions (from early stages of the disease) was another key challenge. Even though we analysed more sections from such lesions to increase the number of fully intact AISs, the number of lesions and in particular the number of patients still turned out to be insufficient to conclude about pyramidal neurons AIS length in active lesions, which will require further studies. As we included some distally cut (i.e. by cryostat sectioning) AISs to our two-dimensional gap analysis (as long as the axon hillock and its corresponding cell body were clearly identifiable with either the SMI32 or the Calbindin labelling), more soma-AIS gap than AIS length measurements could be performed in each tissue category. Finally, despite the limited population size of AISs that could be analysed, the use of a powerful statistical analysis (with linear mixed-effect models) to take the intra-individual variability into account, allowed us to demonstrate significant differences with respect to structural characteristics of AISs, in MS lesions compared with control tissue.

Similar AIS phenotypes (for altered AIS location) were found in both neocortical pyramidal neurons and Purkinje cells in MS grey matter demyelinated lesions, suggesting that a common molecular mechanism may be at play in both cell types. The AIS has been found to be altered in different ways in many pathological conditions.^38–40^ Yet, the mechanisms responsible for these changes remain largely elusive. On the one hand, a calpain-dependent mechanism has been shown to cause AIS dismantling through AIS constituent protein degradation.^25^ On the other hand, a calcium- and calmodulin-dependent protein phosphatase calcineurin has been shown to mediate changes in soma-AIS gap, with the involvement of phosphorylated myosin light chain, an activator of contractile myosin II.^41,42^ Interestingly, calcineurin activity also mediates activity-dependent control of AIS length,^43^ suggesting that it may be a common target of upstream mechanisms to change both soma-AIS gap and AIS length. However, the mechanisms responsible for calcineurin activation and for mediating calcineurin activity still need to be elucidated.

The fact that we observed these soma-AIS gap alterations in both active and inactive demyelinated MS lesions suggests that it is not a late feature of chronically demyelinated axons or degenerating neurons but that it might occur early in the lesion process (noteworthy, no morphological characteristic feature of neurodegeneration was detected in analysed neurons, which however does not rule out the possibility that some may have started undergoing axonal degeneration from a distal location). The detection of longer soma-AIS gaps in active and significantly even longer in inactive cortical lesions could suggest that, even though both inflammation and demyelination are involved in the increased soma-AIS gap phenotype, demyelination per se or the extent of demyelination is likely to play a more prominent role in altering the soma-AIS gap. Yet this demyelination-only effect was not observed in Purkinje cells, as there was no significant difference between active and inactive lesions for soma-AIS gap, suggesting either a different mechanism or different demyelinated lesion characteristics between the two regions. Interestingly, cortical inactive lesions seem to have a synergistic or additive effect on pyramidal neurons’ excitability, which is increased by both an increased soma-AIS gap and an increased AIS length (which was shown by previous modelling studies to increase neuronal excitability^28,31^). It is also interesting to note that our results, suggesting that both inflammation and demyelination are involved in the increased AIS-soma gap phenotype, differ from those obtained in MS animal models (EAE and cuprizone models), where disruption of AIS integrity was shown to be the consequence of inflammation (EAE model) but not demyelination (cuprizone model),^27^ whereas another study showed that demyelination alone (cuprizone model) caused a soma-AIS gap reduction.^28^ It is however difficult to compare human and experimental data, as on the one hand, the duration of the disease is quite different and both inflammation and demyelination are present in MS on the other hand. Further studies will thus be required to shed a light on the mechanisms by which AISs are differently altered in MS, as well as in animal models of MS.

Changes in the soma-AIS gap can have a number of opposing effects on spike initiation, depending on the characteristics of the soma and the AIS (reviewed by Goethals and Brette^31^). Biophysical analyses and modelling suggested that small displacements of an AIS with a fixed number of Nav channels generally cause a decreased AP threshold as the soma-AIS gap increases^34,44,45^ in accordance with results obtained in neocortical pyramidal cells showing that a decrease in soma-AIS gap depolarized AP threshold.^28^ In order to quantitatively assess the effects of the soma-AIS gap changes in MS lesions, and to take the demyelination of MS lesions into account, we simulated computational models of a pyramidal cell and a Purkinje cell-based detailed morphological reconstructions. The overall effect of the modelled AIS changes in both pyramidal neurons and Purkinje cells showed a slight decrease in AP voltage threshold, and we found that demyelination per se did not have a profound effect. These small changes could nevertheless alter the relationship between synaptic input and cell firing and potentially lead to neurological symptoms. However, we assumed here that other parameters such as Nav and Kv channel densities in the AIS remain unchanged. Yet, given what occurs at and around nodes of Ranvier in demyelinated tissue (in both animal models and MS tissue), i.e. redistribution of nodal Nav and Kv isoforms along the axon together with changes in Nav and Kv isoforms expressed in the nodal region,^10,11^ Nav and/or Kv isoform expression or distribution changes may also occur at or around the AIS in active and/or inactive lesions, which may have an additional important impact on the neuron’s excitability properties. Further studies of such potential modifications, together with their chronology relative to nodal changes, are therefore required to better understand how neuronal function is affected, in addition to the effects caused by the AIS structural changes observed in the current study (Fig. 4). We also limited our modelling analysis to the effects soma-AIS gap changes because of their consistency, but did not, for simplicity reasons, take into account the increased AIS length observed only in pyramidal neurons of inactive lesions (but which could not be excluded to take place also in active lesions, as mentioned above). This length change may have additive effects, as previous modelling studies suggested^28,31^). Finally, for pyramidal neurons for instance, such AIS distal shifts may allow GABAergic chandelier synapses aligned along the AIS, by keeping their position, to have a more efficient inhibitory effect on the pyramidal neuron’s excitability.^46^ Again, further studies will thus be necessary to address more comprehensively the changes occurring at the AIS in MS tissue and their impact on neuronal function and disease progression.

## Conclusion

The frequency and functional impact of MS grey matter lesions on disease progression is now well established.^6,36^ Here, we show for the first time that the AIS position is affected in MS lesions, and that this change could play a role in functional alterations. These findings highlight the fact that not only nodes of Ranvier but also AISs can contribute to altered neuronal function and neurodegeneration observed in MS. Finally, the mechanisms of how and when AIS alteration may contribute to the disease development and progression remain to be elucidated.

## Supplementary Material

fcac284_Supplementary_Data

## Abbreviations

AIS =: axon initial segment
AnkG =: ankyrinG
AP =: action potential
BF =: bright field
EAE =: experimental allergic encephalomyelitis
Kv =: voltage-gated potassium channels
LFB =: luxol fast blue
MHC =: major histocompatibility complex
MOG =: myelin oligodendrocyte glycoprotein
MS =: multiple sclerosis
NAGM =: normal-appearing grey matter
Nav =: voltage-gated sodium channels
PLP =: proteolipid protein
ROI =: region of interest
