# Supplementary material for: Alterations of the axon initial segment in multiple sclerosis grey matter
Source: Brain Commun. 2022 Nov 4;4(6):fcac284. doi: 10.1093/braincomms/fcac284 (PMC9700164; doi:10.1093/braincomms/fcac284)
Supplement: fcac284_Supplementary_Data [file fcac284_supplementary_data.pdf]

SUPPLEMENTARY MATERIAL

Model description

Neocortical pyramidal cell. We made the following minor modifications to the neocortical pyramidal cell model[1] for the sake of simplicity: we approximated the AIS geometry to three linear segments (that closely follow the AIS approximately piecewise linear decrease in diameter), defined by the four following diameters along the AIS: $d(l_0) = 5$ µm, $d(l_1) = 2$ µm, $d(l_2) = 1.7$ µm, and $d(l_3) = 1.6$ µm, with $l_0 = 0$ µm (proximal end of the AIS), $l_1 = 0.55\ l_{AIS}$, $l_2 = 0.95\ l_{AIS}$, and $l_3 = l_{AIS}$ (distal end of the AIS).

The model includes a single Nav channel and several types of Kv channels with Hodgkin-Huxley-like kinetics. The main parameters are as follows (please refer to [1] for additional detail): $E_{Na} = 55$ mV, $g_{Nav;D/S} = 60$ pS/µm^2 in dendrites and soma and $g_{Nav;NoR} = 2500$ pS/µm^2 in nodes of Ranvier (intermodal distance: 60 µm). Three types of Kv channels describe (i) a high-voltage-activated channel ("Kv"), (ii) a faster low-voltage activated Kv1-like channel ("Kv1"), and (iii) a slowly-activating and non-inactivating M-type channel ("Km"). $E_K = -85$ mV. Peak conductances (identical for dendrites and soma) are: $g_{Kv;D/S} = 20$ pS/µm^2, $g_{Kv1;D/S} = 100$ pS/µm^2, and $g_{Km;D/S} = 5$ pS/µm^2. In order to avoid hypotheses about the precise spatial distribution of ion channels in the AIS, we distributed the identical number of ion channels as in the original model on the simplified AIS (cone + tube), with constant density along the AIS: $g_{Nav;AIS} = 2917$ pS/µm^2, $g_{Kv;AIS} = 86$ pS/µm^2, $g_{Kv1;AIS} = 161$ pS/µm^2, and $g_{Km;AIS} = 6.3$ pS/µm^2. $g_{Kx;NoR} = g_{Kx;AIS}$. A hyperpolarization-activated current is implemented by I_h channels distributed in the soma and dendrites with an exponential increase in density with distance from the soma. The passive electrical properties of the reconstructed cell were set as follows: $C_m = 0.9$ µF/cm^2, $R_i = 100$ Ωcm, and $R_m = 15$ kΩcm^2.

Purkinje cell. In the Purkinje cell model[2] the geometry of the AIS is not based on a morphological reconstruction but represented by a single segment of constant diameter. We did the following minor modifications to the AIS geometry: while keeping the original AIS length (21 µm), we changed the AIS diameter (to 1.94 µm) to accommodate for a larger fraction of Nav channels at the AIS.

The model contains Na^+, K^+, and Ca^{2+} conductances with several subtypes for Kv, Ca-dependent potassium (Kca), and calcium (Ca) channels based on Markovian or Hodgkin-Huxley-like dynamics[2]. It furthermore contains a mixed cationic channel (HCN1) as well as explicit dynamics for the internal calcium buffer[2]. The model for the Nav channel is based on a Markovian state dynamics and accounts for transient, persistent and resurgent Na^+-current

components[3]. E_{Na} = 75 mV. We kept the same total number of Nav channels as in the original model but distributed them slightly differently to reduce the ratio of somatic to AIS Nav channel density. Peak conductances were: $g_{Nav;D}$ = 160 pS/µm^2 in dendrites, $g_{Nav;S}$ = 1861 pS/µm^2 in the soma and $g_{Nav;AIS}$ = 8095 pS/µm^2 in the AIS ($g_{Nav;S}/g_{Nav;AIS}$ ~ 0.23 as opposed to ~0.43 in the original model); $g_{Nav;NoR}$ = 300 pS/µm^2 in nodes of Ranvier (internodal distance: 100 µm). We furthermore distributed the AIS Kv channels homogenously along the AIS while keeping the average channel density at the AIS constant, leading to $g_{Nav;AIS}$ = 3948 pS/µm^2, $g_{Kv1.1;AIS}$ = 12 pS/µm^2 , and $g_{Kv3.4;AIS}$ = 49 pS/µm^2. All other parameters were unchanged. Passive electrical properties were set as follows: C_m = 0.77 µF/cm^2, R_i = 122 Ωcm, and R_m = 0.91 kΩcm^2. For all other parameters, please refer to [2].

Simulation protocols

Modification of AIS morphology. We systematically varied the soma-AIS gap and AIS channel densities as follows. To mimic a finite soma-AIS gap, of length l_{gap}, we set channel densities along this soma-AIS gap to their somatic values and accordingly extended the AIS distally at constant diameter on a length l_{gap} to preserve the total AIS length.

Myelination and demyelination (pyramidal cell). Kole et al. originally mimicked myelination by reducing C_m to 0.02 µF/cm^2 along the internodal sections[1]. Since effective channel conductances might have to reflect the high resistance imposed by a myelin sheath, we decided to additionally suppress ion channels in myelinated internodes (setting peak conductance values to zero). Conversely, demyelination was mimicked by restoring the internodal membrane capacitance to the default value C_m = 0.9 µF/cm^2 and by setting channel peak conductances to finite values that were determined as follows. Based on the observation that ion channels are redistributed in the membrane of axons upon disorganized nodes of Ranvier in demyelinated MS lesions[4, 5], we considered peak conductances of demyelinated internodes and within the nodes to be given by the approximate mean value of the respective somatic and node peak conductances: $g_{Nav;dem}$ = 106 pS/µm^2, $g_{Kv;dem}$ = 41 pS/µm^2, $g_{Kv1; dem}$ = 22 pS/µm^2, and $g_{Km; dem}$ = 5.5 pS/µm^2.

Myelination and demyelination (Purkinje cell). In line with the original model, we mimicked myelination by a vanishing C_m and vanishing ionic conductances along the internode. In our study, we mimicked demyelination by restoring the internodal membrane capacitance to the default value C_m = 0.77 µF/cm^2. We furthermore reduced channel concentrations within the nodes to the average densities between internodes and nodes in myelinated conditions. For Nav, we thus obtained $g_{Nav;dem}$ = 13 pS/µm^2.

Determination of the voltage threshold. Because we were mainly interested in the impact of AIS morphological parameters on the voltage threshold, the procedure used to determine a voltage threshold V_{thr} for APs recorded in the soma is the following: whenever the time derivative of the somatic voltage $\mathrm{d}V(t)/\mathrm{d}t$ crosses a fixed threshold c, the voltage threshold is given by the somatic potential at the time t_c of threshold crossing, $V_{\text{thr}} = V(t_c)$. In the case of multiple threshold crossings, V_{thr} did not vary between APs. While the absolute values of V_{thr} depend slightly on the choice of c, the relative variation does not, nor did the number of threshold crossings within a reasonable range. Throughout this study, we used $c = 20$ mV/ms.

Current injection. To elicit stationary firing in the pyramidal cell, a constant current of 0.9 nA was injected in the soma during the simulation. The Purkinje cell was spontaneously active, thus no current was injected.

References

1. Kole MHP, Ilschner SU, Kampa BM, Williams SR, Ruben PC, Stuart GJ. Action potential generation requires a high sodium channel density in the axon initial segment. *Nat Neurosci*. 2008;11(2):178-186.

2. Masoli S, Solinas S, D'Angelo E. Action potential processing in a detailed Purkinje cell model reveals a critical role for axonal compartmentalization. *Front Cell Neurosci*. 2015;9.

3. Raman IM, Bean BP. Inactivation and Recovery of Sodium Currents in Cerebellar Purkinje Neurons: Evidence for Two Mechanisms. *Biophys J*. 2001;80(2):729-737.

4. Coman I, Aigrot MS, Seilhean D, et al. Nodal, paranodal and juxtaparanodal axonal proteins during demyelination and remyelination in multiple sclerosis. *Brain*. 2006;129(12):3186-3195.

5. Craner MJ, Newcombe J, Black JA, Hartle C, Cuzner ML, Waxman SG. Molecular changes in neurons in multiple sclerosis: Altered axonal expression of Nav1.2 and Nav1.6 sodium channels and Na+/Ca2+ exchanger. *Proc Natl Acad Sci*. 2004;101(21):8168-8173.

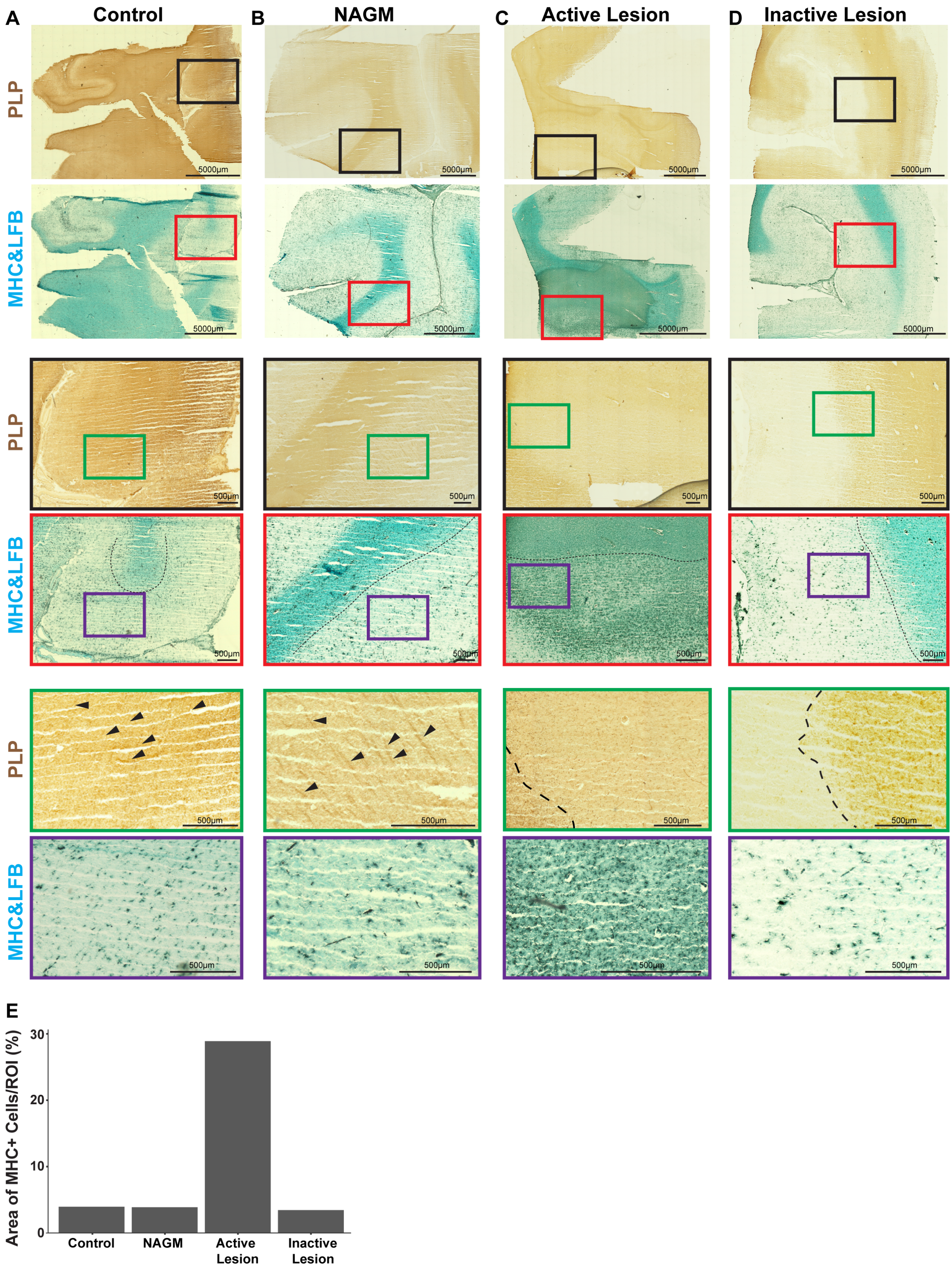

A
Control
B
NAGM
C
Active Lesion
D
Inactive Lesion
PLP
MHC&LFB
5000μm
500μm
E
Area of MHC+ Cells/ROI (%)
30
20
10
0
Control
NAGM
Active Lesion
Inactive Lesion

Supplementary Fig. 1: Characterization of Cortical MS lesions

Bright field tile images of PLP labeling and LFB & MHC labeling acquired with a 5x objective in the cortex of (A) a control (B) an NAGM (C) an active lesion (D) and an inactive lesion section. Higher magnification of the selected regions from PLP (indicated by the black frames) and LFB & MHC (indicated by the red frames) are presented (dashed lines in the magnified red frames represent the white matter – grey matter border). Further magnification of the selected sub-regions from these frames, indicated by the green and purple frames for PLP and LFB & MHC labeling respectively are also presented for better visualization. Arrowheads show the "stripe-like" myelin fibers labeled with PLP in control and NAGM panels and dashed lines represent the lesion border in active and inactive lesion panels (E) Percentage area of MHC+ cells per ROI (corresponding to the purple frame).

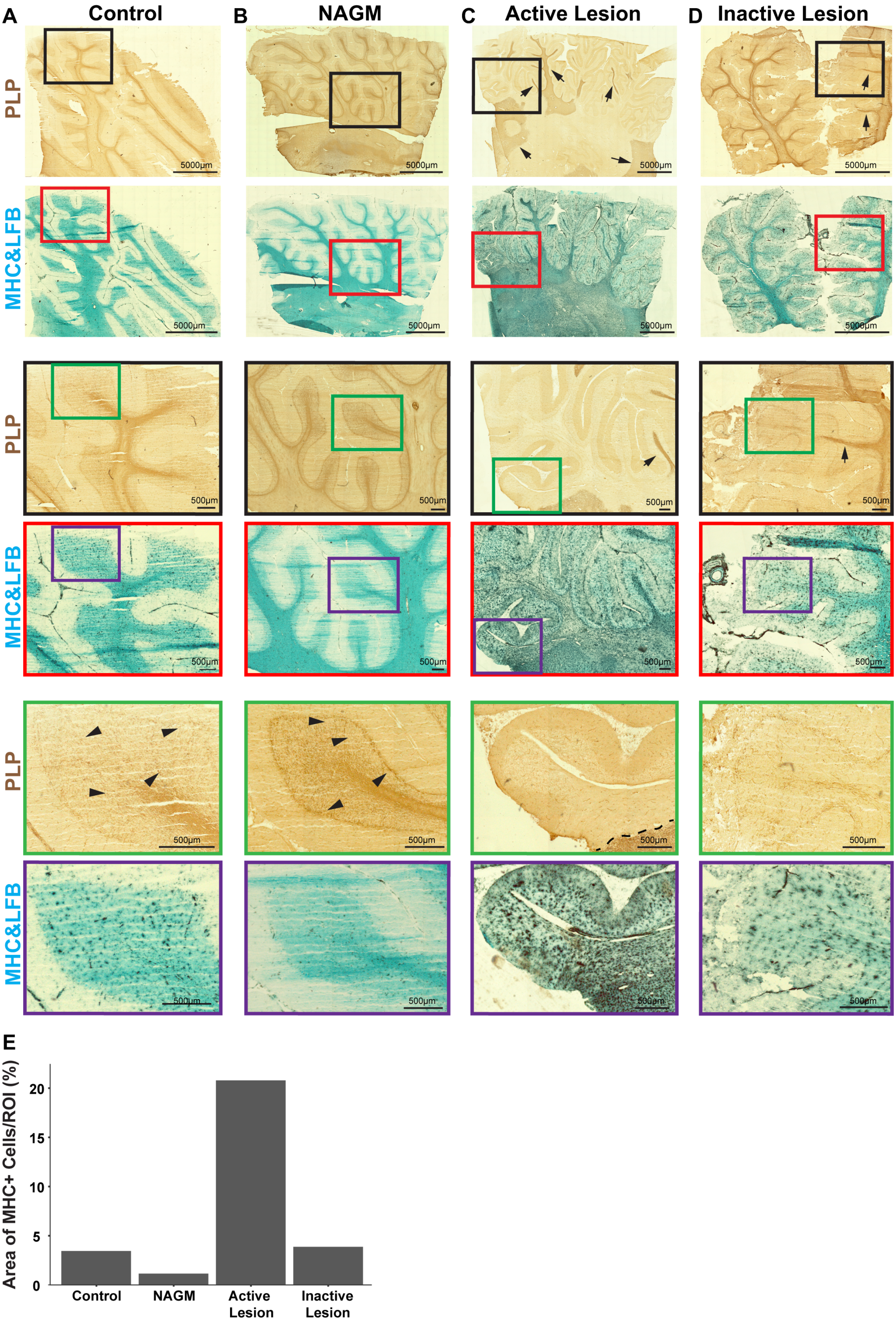
A
Control
B
NAGM
C
Active Lesion
D
Inactive Lesion
PLP
MHC&LFB
5000μm
500μm
E
Area of MHC+ Cells/ROI (%)
0
5
10
15
20
Control
NAGM
Active Lesion
Inactive Lesion

Supplementary Fig. 2: Characterization of Cerebellar MS lesions

Bright field tile images of PLP and LFB & MHC labeling acquired with a 5x objective in the cerebellum of (A) a control (B) an NAGM (C) an active lesion (D) and an inactive lesion section. Higher magnification of the selected regions from PLP (indicated by the black frames) and LFB & MHC (indicated by the red frames) are presented. Further magnification of the selected sub-regions from these frames, indicated by the green and purple frames for PLP and LFB & MHC labelings' respectively are also presented for better visualization. Arrowheads show the "stripe-like" myelin fibers labeled with PLP in control and NAGM panels, arrows show the remaining myelinated white matter tracts in almost fully demyelinated selected regions, the dashed line represents the lesion border in the active lesion panel while no border is represented for inactive lesion panel as the selected area is fully demyelinated. (E) Percentage area of MHC+ cells per ROI (corresponding to the purple frame).

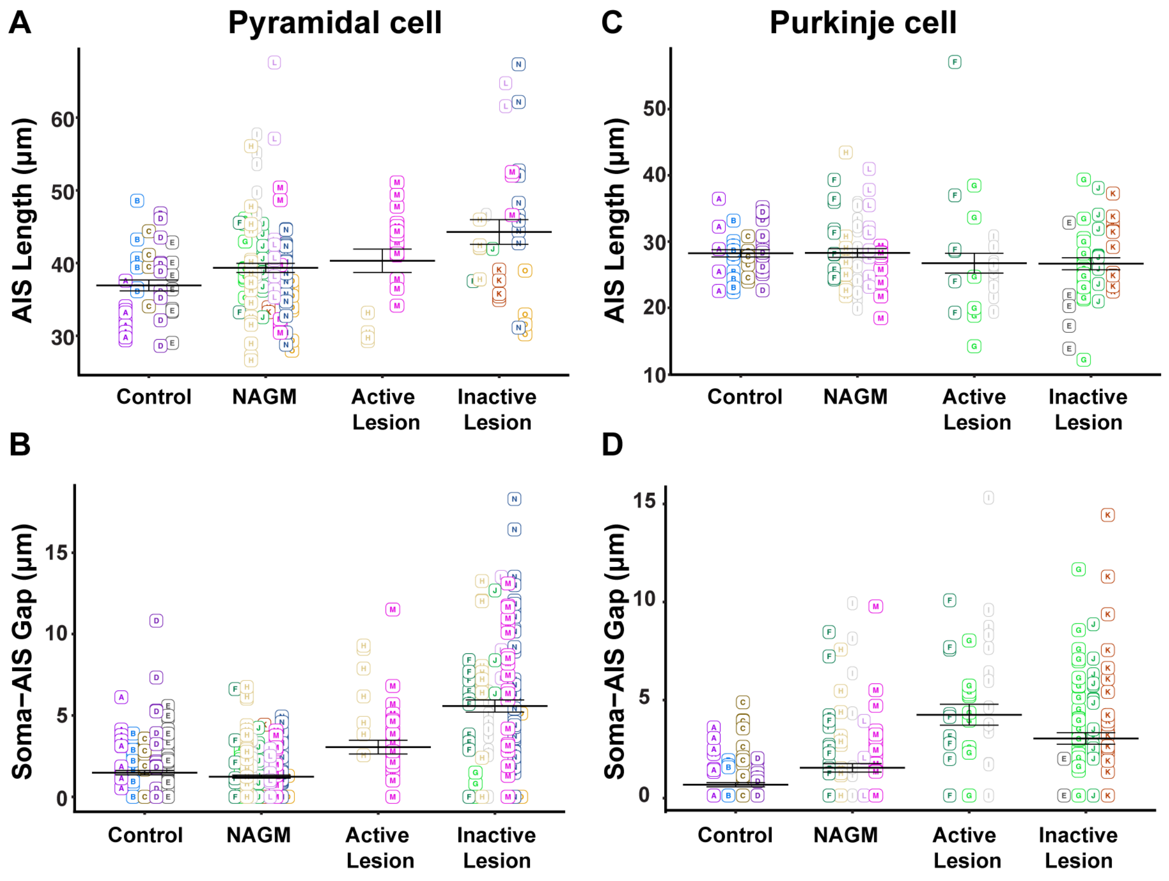

Supplementary Fig. 3: AIS length and soma-AIS gap measures from pyramidal neurons and Purkinje cells plotted as a function of the patient of origin

AIS length (A and C) and soma-AIS gap measures (B and D) from pyramidal neurons and Purkinje cells were plotted as a function of the patient of origin: each AIS is represented by a square, and the patient the AIS is coming from is defined by the square's letter and color. AISs coming from the same patient are grouped at the same position along the x axis

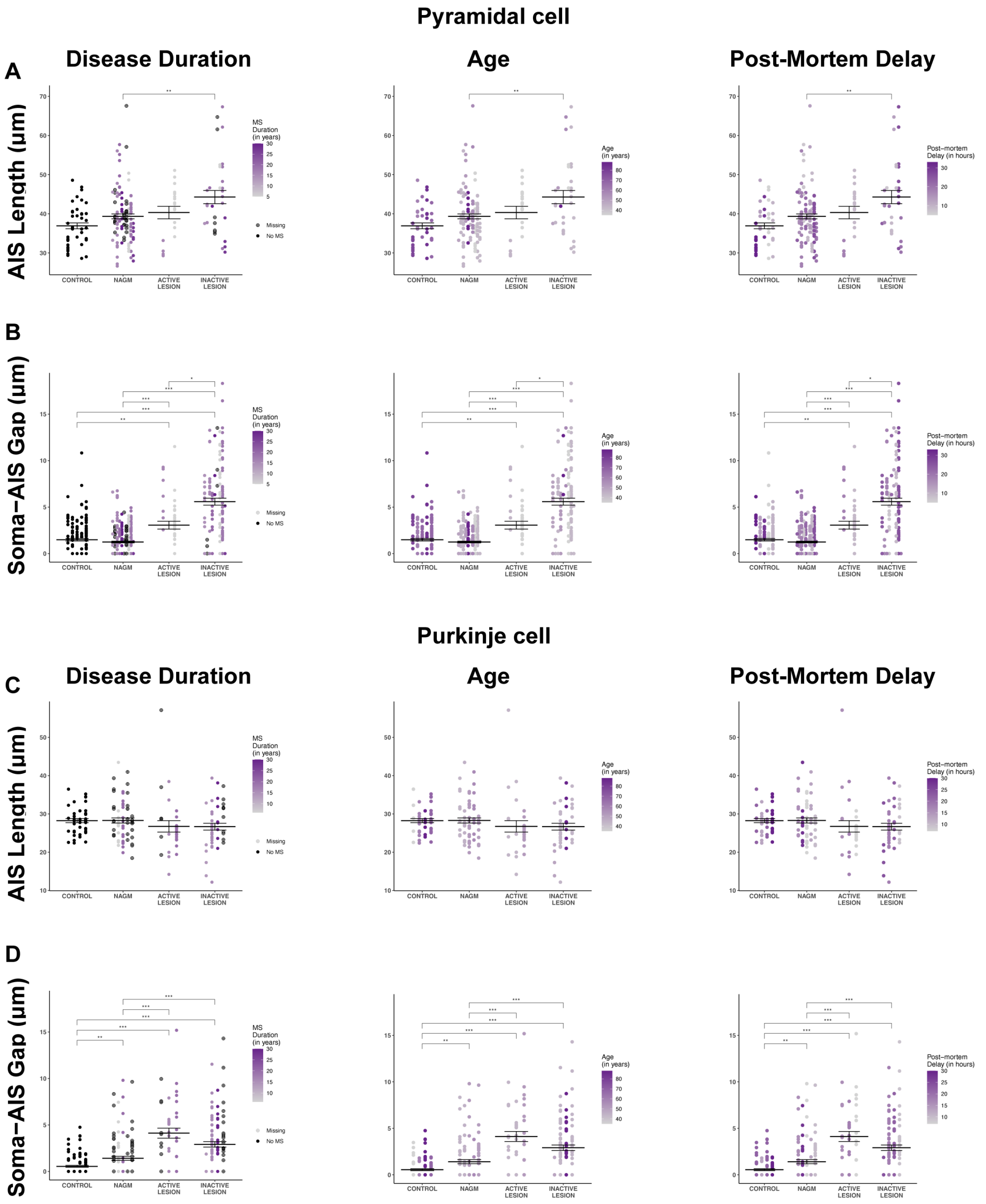

Supplementary Fig. 4: AIS length and soma-AIS gap measures plotted as a function of disease duration, age of the patient and post-mortem delay of human samples

Graphs representing AIS length (A and C) and soma-AIS gap (B and D) measures from pyramidal neurons and Purkinje cells as a function of disease duration, age of the patient and post-mortem delay before the brain sample was collected and frozen (as reported in Table 1). Each dot represents a different AIS.
